# The role of bile acid in intestinal metaplasia

**DOI:** 10.3389/fphys.2023.1115250

**Published:** 2023-02-20

**Authors:** Menglei Wang, Enzhe Lou, Zengfu Xue

**Affiliations:** Department of Digestive Diseases, The First Affiliated Hospital of Xiamen University, Xiamen University, Xiamen, China

**Keywords:** bile acid, gastroesophageal reflux, intestinal metaplasia, Barrett’s esophagus (BE), molecular mechanism

## Abstract

A precancerous lesion of gastric cancer (GC), intestinal metaplasia (IM) is a pathological transformation of non-intestinal epithelium into an intestinal-like mucosa. It greatly raises the risk of developing the intestinal type of GC, which is frequently observed in the stomach and esophagus. It is understood that esophageal adenocarcinoma’s precursor lesion, chronic gastroesophageal reflux disease (GERD), is what causes Barrett’s esophagus (BE), an acquired condition. Recently, Bile acids (BAs), which are one of the compositions of gastric and duodenal contents, have been confirmed that it led to the occurrence and development of BE and gastric intestinal metaplasia (GIM). The objective of the current review is to discuss the mechanism of IM induced by bile acids. This review serves as a foundation for further research aimed at improving the way BE and GIM are currently managed.

## Introduction

BE, as a disorder that grows from a chronic inflammatory environment (esophagitis) caused by GERD, is essentially an acquired metagenesis abnormality in which the normal stratified and non-keratinizing squamous epithelium is substituted by metaplastic columnar epithelium. BE is significant in medicine mainly because those who have it have a 400-fold increased risk of developing esophageal cancer compared to the general population (Chai and Jamal, 2012). EAC is a fatal malignancy with an average 18% 5-year overall survival rate (Siegel et al., 2021). In the last few decades, the significance of gastric acid reflux exposed in the pathology of EAC was overemphasized before the use of proton pump inhibitors failed to reduce the increasing number of disease cases. Furthermore, in the rat reflux model, stomach acid was unable to cause BE to form on its own. As a result, acid reflux is not the only component contributing to the development of GERD into EAC. Numerous investigations have recently demonstrated that BAs, a different component of the contents of duodenogastric reflux (DGR), are connected to the emergence and progression of BE (Souza et al., 2008).

GC remains one of the most important cancers worldwide, ranking fifth for incidence and fourth for mortality globally (Sung et al., 2021). The Correa model describes how intestinal gastric cancer develops in stages and sequentially: superficial gastritis, atrophic gastritis, IM, dysplasia, and carcinoma. In general, chronic environmental inflammation is the primary cause of GIM (Correa, 1988; Correa et al., 2004). Certain types of chronic proinflammatory factors, such as inflammation linked to *Helicobacter pylori* (Hp) infection, bile acid reflux, cigarette smoking, radiation exposure, alcohol consumption, and a diet deficient in fruits, vegetables, and vitamins, can trigger or accelerate this inflammation (Tatsugami et al., 2012; Choi et al., 2018). According to a multi-centric and large-scale cross-sectional study in Japan, high concentrations of BAs are related to an increased risk of GIM regardless of *Hp* infection (Matsuhisa et al., 2013). And the eradication of *Hp* cannot reduce the risk of GC in patients with IM. It is commonly acknowledged that BAs play a significant role in the establishment of GIM. One of the main constituents of bile and DGR, BAs are a category of steroid acids with distinctive physical, chemical, and biological properties. The most abundant BAs in the patient’s DGR are deoxycholic acid (DCA) and chenodeoxycholic acid (CDCA) (Peng et al., 2014). DCA, as an unconjugated bile acid, is linked to BE and GIM development. Despite the fact that BAs are widely known to play a crucial part in the formation of GIM and BE (Yu et al., 2019), the molecular mechanisms underlying the initiation and progression of IM remain unclear (Shen et al., 2016).

Over the past few decades, more research has been done to better understand the pathophysiology of BE and GIM caused by BAs. Previous studies have shown that BAs drive the formation of IM through various signaling pathways and downstream transcription factors, including CDX2, KLF4, HNF4α, FOXP3, FXR, microRNA, and so on (Thompson et al., 2018). Furthermore, the role of BAs on the crosstalk between macrophages and other epithelial cells in chronic inflammation during IM should be focused on. Herein we review the IM of the esophagus and stomach induced by BAs, and discuss their potential specific mechanisms.

## The classification and pathology of bile acids

According to the source, BAs are divided into primary bile acids and secondary bile acids, primary bile acids including cholic acid (CA) and CDCA in humans. When it comes to carcinogenesis, different bile acids have different biological effects, and their specific effects may be related to their chemical structure and hydrophobic characteristics. The damage to gastric mucosa is mainly caused by hydrophobic bile acids. Hydrophilic bile acids have been shown to have cellular protective effects (Goldman et al., 2010). When the patient receives treatment for long-term acid suppression, the stomach’s pH decreases in acidity (pH4-6), which allows the intestinal bacterial flora to spread up to the stomach (Williams, 2001) and improves the ability of bacterial de-conjugation, finally increases the content of unconjugated bile acids in the bile acids pool (Hofmann and Mysels, 1992; Theisen et al., 2000). Since unconjugated bile acids are more lipophilic than conjugated ones, they can be simply diffused through the brush boundary of enterocytes and quickly absorbed. Shen *et al.* used DCA-induced esophageal squamous epithelial cell line Het-1a to reprogram to express intestinal epithelial cell markers, CDX2 and MUC2 (Shen et al., 2016). Additionally, Huo et al. discovered that whereas hydrophobic UDCA did not harm the cellular DNA of the BE cell lines, DCA induced an oxidative stress reaction and cell DNA damage (Huo et al., 2011). As a result, ingestion of hydrophilic bile acids such as UDCA to alter bile acid composition may have a therapeutic effect on IM (Marteau et al., 1990).

## Transcription factors in IM: CDX1, CDX2

There are three vertebrate homologs of cauda (CDX1, CDX2, and CDX4 in humans). Although neither CDX1 nor CDX2 is normally expressed in the human stomach or esophagus, they are in the epithelium of the large and small intestines. However, both GIM and BE exhibit abnormal expression of them. Kazumori *et al.* found that BAs activate the expression of CDX1 in immature esophageal keratinocytes and illustrated how CDX1 interacts with CDX2 and how that interaction works, stimulating the development of BE by binding directly to each other’s promoters, leading to upregulation of BE (Kazumori et al., 2009). Due to the many CDX responsive sites present in the CDX promoter, CDX1 and CDX2 can also bind to their own promoter and use an efficient auto-regulatory loop to drive their own expression (Kazumori et al., 2009).

In intestinal epithelial cells, CDX1 promotes differentiation and functions as a component of the transcriptional network that grants embryonic stem cells pluripotency. Mutoh *et al.* observed causal relationship between ectopic CDX1 and GIM by observing that transgenic expression of CDX1 is sufficient to cause IM in the stomach of transgenic mice (Mutoh et al., 2004a). Park *et al.* found that BAs induce the gene expression of cyclooxygenase-2(COX-2) which is associated with GIM *via* the induction of CDX1 (Park et al., 2008a). The microarray data also demonstrated elevated CDX1 and CDX2 expression in BE (Harada et al., 2003). In esophageal epithelial cells, BAs elevated CDX1 promoter activity and CDX1 protein in a dose-dependent manner, according to Kazumori et al. (van den Brink et al., 2002). CDX1 can be transactivated by a variety of different signaling mechanisms, including Wnt/β-catenin signaling (Prinos et al., 2001). SALL4, a zinc-finger transcription factor, is induced by CDX1 to positively regulate OCT4, c-MYC, SOX2, KLF4 and KLF5 (Zhang et al., 2006). Through the transcriptional route that CDX1 has established, dedifferentiated cells can be transdifferentiated into intestinal epithelial cells and gastric epithelial cells can be guided toward a less differentiated intestinal stem/progenitor-like state. A crucial intestine-specific transcription factor (TF), CDX2 is involved in the formation, proliferation, and differentiation of intestinal epithelial cells as well as the maintenance of the intestinal phenotype. Numerous investigations have demonstrated that GIM and BE commonly exhibit CDX2 up-expression (Kazumori et al., 2006). It is thought that ectopic CDX2 expression gone awry in the upper gastrointestinal tract is a crucial step in the pathophysiology of both BE and GIM. In any case, the ability to express CDX2 may be a molecular trigger for intestinal-type metaplasia’s emergence (Burke and Tosh, 2012). Silberg *et al.* observed that intestinal metaplasia occurs in mouse models with CDX2 expression directed to the glandular stomach, along with the loss of stomach characteristics and the acquisition of intestine characteristics, such as the expression of MUC2 and the existence of intestinal-type goblet cells. Previous studies have shown that activated CDX2 promotes IM by stimulating the transcription of intestinal markers involved in cell cycle progression, proliferation, differentiation and intestinal cell fate specification including MUC2, KLF4, VILLIN1, glucagon, guanylyl cyclase, claudin-3 and-4, SHH and trefoil factor 3 (TFF3) (Mutoh et al., 2004b; Satake et al., 2008; Mutoh et al., 2010; Barros et al., 2012).

In previous studies, DCA showed the strongest effect on CDX2 transcription of all BAs (Hu et al., 2007). One urgent question to be addressed is how BAs activate CDX2 in gastric epithelium. Deciphering the upstream networks that result in CDX2 ectopic expression is crucial (Niu et al., 2017). There are two main kinds of pathways that could be imagined as being involved in this process: 1) signaling pathways that are normally unavailable from the stomach and esophagus but involved in intestinal differentiation and formation that become active in the stomach and esophagus under particular circumstances, and 2) signaling pathways that are involved in establishing and maintaining the gastric and esophageal phenotype but are inactivated (Barros et al., 2012). So, it seems to reason that CDX2 would be regulated in the stomach both negatively and positively. As a result, it is expected that exposure to BAs in the normal mucosa will reduce the negative regulatory forces and raise the positive regulatory forces, tipping the balance in favor of CDX2 expression (Barros et al., 2012). By activating transcription factors and signaling pathways in the complex environment where IM takes place, BAs-induced CDX2 can be positively controlled (Niu et al., 2017). As mentioned by Lu *et al.* in their study, BAs-induced IM promotes CDX2 and intestinal specific makers up expression through the miR-92a-1-5p/FoxD1/NF-κB, the FXR/SHP/NF-κB, and FoxO4/CDX2 pathways (Lu et al., 2021). BAs-induced HNF4α, NF-κB, CDX1 and so no, all regulate the CDX2 promoter activity or the expression of CDX2 (Kazumori et al., 2009; Yu et al., 2019; Wang et al., 2021). For example, Kazumori *et al.* found that the activation of NF-κB by BAs causes esophageal keratinocytes to express more CDX2 (Kazumori et al., 2006). Additionally, proinflammatory IL-6 in gastric cells can stimulate the SHP-2/ERK/MAPK pathway, which in turn regulates CDX2. SHP is also a member of the downstream signaling pathway of FXR. FXR upregulates the expression of SHP, which further inhibits the synthesis of BAs. The BA-induced down-expression of SOX2 and FOXD1 leads to CDX2 upregulation (Li et al., 2019; Yuan et al., 2019). Furthermore, SOX2 inhibits the activity of other transcription factors in intestinal cells, negatively modulating the CDX2 promoter (Asonuma et al., 2009).

## Transcription factors in IM: KLF4, KLF5

Humans have about 20 members of the KLF family, which is structurally distinguished by three tandem zinc-finger domains at the C-terminus. Numerous biological processes, like apoptosis, terminal differentiation, and proliferation, are regulated by KLF4 (Yan et al., 2016). The colonic epithelium of KLF4^−/−^ animals reveals patchy expression of the goblet cell marker MUC2, a substantial drop in goblet cell quantity, aberrant goblet cell shape, and abnormal goblet cell expression (Katz et al., 2002). Kazumori *et al.* found that KLF4 expression was strongly observed in BE and its expression is induced in response to BAs (Kazumori et al., 2011). When KLF4 expression is positively regulated by BAs, even if the induction level is low, its self-replication process will likely lead to increased expression of KLF4 (Kazumori et al., 2011). This is because KLF4 may positively regulate its own promoter activity. According to reports, KLF4 and CDX2 have similar roles in the growth and development of the intestinal mucosa (Ton-That et al., 1997). KLF4 and CDX2 may upregulate one another and encourage the expression of columnar genes in IM (Chen et al., 2017). Yan et al. also discovered that DCA costimulates the BMP4 pathway and KLF4 and that BMP4 can upregulate KLF4, CDX2, MUC2, and MUC5ac expression. These findings imply that KLF4, which supports the phenotypic change of a mature esophageal squamous epithelium by up-regulating KLF4, should be situated downstream of BMP4 pathways. KLF4 expression is suppressed in the gastrointestinal tract by Notch signaling. Stopping Notch signaling Through KLF4’s ICN-responsive components, Notch encourages KLF4 expression and the start of a transdifferentiating pathway that leads to BE-like metaplasia (Vega et al., 2014; Yan et al., 2016).

KLF5 also known as intestine-enriched Krüppel-like factor (IKLF) and BTEB2. KLF5 has been shown to play a part in biological processes such as intestine development, cardiovascular remodeling, adipogenesis, and embryonic development *in vivo* models. KLF5 is abundantly expressed throughout development in the epithelium of the intestine, colon, and stomach in both humans and mice (Dong and Chen, 2009). KLF5’s ability to replace KLF4 and produce inducible iPS cells demonstrates how it contributes to the development of stemness (Nakagawa et al., 2008). Xia *et al.* found that DCA mediated the intestinal transdifferentiation of the esophageal squamous epithelium in a KLF5-dependent manner (Xia et al., 2019), suggesting that KLF5 is linked to an increased risk of BE development. Fujii et al. discovered that CDX1 induces SALL4 and KLF5 in gastric epithelial cells to give them an intestinal character (Fujii et al., 2012). The findings above imply that KLF5 is crucial to BAs-induced IM.

## Transcription factors in IM: HNF4α

The highly conserved nuclear receptor hepatocyte nuclear factor-4α(HNF4α), which is expressed in the intestine throughout early development, has a role in controlling the growth and functionality of the gut. HNF4α is necessary for goblet cell maturation and normal colon epithelial development. Additionally, HNF4α is not present in the healthy esophagus, but it is expressed and directly causes the columnar phenotype in BE (Colleypriest et al., 2017). Colleypriest et al. also discovered that TFF3 and E-cadherin expression was induced by ectopic expression of HNF4α but not CDX2 (Colleypriest et al., 2017). The up-expression of HNF4α was also observed in GIM and GAC (Tanaka et al., 2006). HNF4α could directly influence CDX2 in the development of the gut, according to prior findings (Boyd et al., 2009). In the last decade of research, it has been demonstrated that HNF4α plays a critical role in mediating the trans-differentiation of gastric epithelial cells in response to BAs exposure. Mechanically, DCA treatment could activate the TGR5 pathway and cause HNF4α expression. In addition, HNF4α can bind to its own promoter and encourage its positive expressions, like KLF4 and CDX2. The HNF4α gene uses two distinct promoters, P1 and P2, both of which are non-redundantly engaged in IM formation. It is interesting to note that a prior study found that P1-HNF4α is considerably upregulated without P2-HNF4α overexpression, indicating that HNF4α isoform splicing is important in BE formation. As a result, at the onset of various disorders, P1- and P2-HNF4α functions and distribution may significantly vary (Green et al., 2014).

## Transcription factors in IM: FOXP3

A crucial transcription factor called Forkhead Box Protein 3(FOXP3) is involved in controlling the growth and operation of regulatory T-cells (Tregs) as well as preserving immunological homeostasis (Wang et al., 2017). According to a recent study, FOXP3 interacts with HDAC6 and HNF4α to generate a loop that contributes to IM brought on by BAs (Zhang et al., 2022). In IM tissues, FOXP3 is also inversely associated with HDAC6 and HNF4α: 1) in HDAC6 overexpression cells, FOXP3 notably reduces in mRNA and protein levels; 2) FOXP3 inhibits the transcription of HNF4α and further inhibits the expression of downstream intestine makers. In addition, FOXP3 also can downregulate the level of MUC2, KLF4, and CDX2. Zhang et al. proposed that FOXP3 may be a potential downstream target that links to HNF4α. In conclusion, the HDAC6/FOXP3/HNF4α loop may be crucial for the growth of IM (Zhang et al., 2022).

## Transcription factors that inhibit IM: SOX2, FoxO4

In contrast to CDX2, SOX2 negatively regulates intestinal differentiation. An HMG-box transcription factor, SOX2, plays a critical role in esophageal and gastric differentiation, which leads to the generation of the stratified squamous epithelium in mice. A delicate balance between SOX2 and CDX2 expression in the digestive tract is necessary for healthy formation, according to reports indicating SOX2 and CDX2 suppressed one another (Raghoebir et al., 2013). In Het-1A cell lines, Shen et al. discovered that treatment to DCA decreased SOX2 expression and enhanced CDX2 expression (Shen et al., 2016). As a result of SOX2 suppression, CDX2 expression was elevated, suggesting that DCA upset the equilibrium between SOX2 and CDX2 and, as a result, promoted a phenotypic shift in the esophageal squamous epithelium. Yuan *et al.* also found that the expression of SOX2 was decreased and the expression of CDX2 was increased in DCA-treated gastric cells (Yuan et al., 2019). Additionally, in CDX2-overexpressing gastric cells, SOX2 reduced the induction of intestine-specific markers (KLF4, HNF4α, and cadherin 17) by CDX2. However, in cells lacking CDX2 expression, there was no direct regulatory connection between SOX2 and those markers. By using a dual-luciferase reporter assay and Co-IP, Yuan *et al.* found that SOX2 could suppress the transcriptional effect of CDX2 on its genomic target sites and they could form SOX2-CDX2 protein complexes in the nucleus, which might be one of the most important mechanisms of SOX2 suppress the downstream DNA-binding capacity of CDX2 (Yuan et al., 2019). Additionally, miR-21, which can be activated by particular bile acid concentrations, prevents SOX2 from being expressed by directly interacting with its 3′-UTR, so easing the inhibition of CDX2 caused by SOX2. The methylation and expression status of CDX2 are both inhibited by SOX2, which is another potential method by which SOX2 reduces CDX2 expression in the gastric mucosa. Niu et al. found that SOX2 knockdown by RNAi in the GES-1 cell line immediately caused CDX2’s promoter to demethylate, which made it easier for CDX2 to express itself at the mRNA level (Niu et al., 2017). Finally, the combination of SOX2 RNAi and CDX2 overexpression effectively induced the phenotype transformation towards GIM in GES-1 cells. Therefore, epigenetic changes can be partly responsible for SOX2 and CDX2’s abnormal expression (Niu et al., 2017). The precise mechanism of SOX2 methylation and SOX2 maintaining the methylation status of the CDX2 promoter, however, remains unknown and needs more research (Niu et al., 2017).

The Forkhead box O(FoxO) transcription factor family contains four related members: FoxO1, FoxO3, FoxO4 and FoxO6 (Daitoku et al., 2011). FoxO4 was previously reported to be a negative regulator in colorectal cancer (Liu et al., 2011). Lu et al. discovered that CDCA-based CDX2 regulation was controlled by potential FoxO4 binding sites and was negatively regulated by p-FoxO4 in gastric cells (Lu et al., 2021). The negative regulatory relationship between CDX2 and p-FoxO4 has also been verified in normal and GIM tissue array. However, the precise mechanism of p-FoxO4 regulated by BA is still unclear. In addition, an earlier investigation had shown that CDCA significantly upregulates CDX2 at both the mRNA and protein levels, and that FoxO4 may be involved in this regulation (Lu et al., 2021). Recent study found that resveratrol, a natural polyphenol with antioxidant, anti-inflammatory, and anticancer properties (Huminiecki and Horbańczuk, 2018), can regulate FoxO4 through the PI3K/AKT pathway, and FoxO4 directly targets the binding of the CDX2 promoter region, thereby inhibiting the expression of CDCA-induced CDX2 (Lu et al., 2021).

## BAs promote IM *via* NF-κB signaling pathway

Proliferation, inflammation, apoptosis, and differentiation are only a few of the significant biological processes in which nuclear factor (NF)-κB is involved. Normal squamous epithelial lining of the esophagus does not have NF-κB activated, whereas GERD-inflamed oesophageal epithelium does (O'Riordan et al., 2005). In esophageal keratinocytes in BE, prior work showed that BAs increase CDX2 transcription and upregulate CDX2 expression *via* NF-κB (Kazumori et al., 2006). Recently, Yu et al. discovered that treatment with PDTC, an inhibitor of NF-κB activation, significantly reduced BAs-induced protein production of CDX2 and that exposure of GES-1 cells to BAs increased NF-κB activity and protein expression as well as the expression of p50 and p65 proteins (Yu et al., 2019). These findings suggested that NF-κB is involved in the control of CDX2 expression generated by BAs. The CDX2 promoter was shown by Kim et al. to include an NF-κB binding site that can be accessed by p50/p65, and a quantitative chromatin immunoprecipitation test revealed that BAs control CDX2 expression by enhancing p50’s binding to the CDX2 promoter but not p65’s (Yu et al., 2019). Huo et al. also discovered that the CDX2 promoter activation by bile salts and acid depends on elevated nuclear p65 protein levels.

There are numerous signals that can activate NF-κB, but they all eventually focus on the cytoplasmic IB-NF-κB-PKAc complex as their common target. Huo *et al.* found acid and bile salts in normal esophageal squamous cells from BE trigger the NADPH oxidase system to produce H2O2, which then activates the IB-NF-κB-PKAc complex through a sequence of phosphorylations to further regulate CDX2 transcription (Huo et al., 2018). FOXD1, FXR, CDX1, KLF4, *etc*., have all been shown to affect NF-κB of expression or activity in the context of IM (Chen et al., 2017).

## BAs promote IM *via* BMP4 signaling pathway

P-Smad1/5/8, Smad4, and BMP4 are some of the transforming growth factor (TGF)-β family’s major subfamilies and downstream targets, respectively (Yan et al., 2016). BMP4 is a critical modulator of the interactions between epithelial and mesenchymal cells in the intestine that result in the development of the intestinal epithelium (Barros et al., 2008). BMP4 was found to be strongly expressed in BE epithelium in a prior serial examination of the gene expression library of BE, but not in squamous epithelium (van Baal et al., 2005). Zhou *et al.* demonstrated that conjugated bile salts at an acid pH significantly upregulated BMP4 expression by triggering CDX2 expression in human esophageal epithelial cells. This finding suggests that BMP4 plays a significant role in the progression of BE (Zhou et al., 2009). Additionally, it was demonstrated that the BMP4 pathway was active in GIM and that it colocalized with abnormal CDX2 expression, confirming the favorable influence of BMP4 on CDX2 expression *in vitro* studies (Barros et al., 2008).

The specific regulatory mechanism of BAs-induced BMP4 to CDX2 maybe, 1) BMP4/SMAD pathway involved in CDX2 regulation (Barros et al., 2008), 2) BMP4 regulates CDX2 by regulating other transcription factors, such as KLF4 (Yan et al., 2016). Wang *et al.* demonstrated that the esophageal squamous epithelium is damaged by acid and bile to secrete SHH, which in turn causes mesenchymal secretion of BMP4, and then BMP4 signaling into the epithelium to activate SOX9, which finally activates the columnar cell transcription program (Wang et al., 2010). Yan et al. discovered that BMP4 upregulates the expression of KLF4, CDX2, MUC2, and MUC5ac and that DCA can activate the BMP4 pathway and KLF4 expression. These findings imply that KLF4, which supports the phenotypic change of a mature esophageal squamous epithelium by up-regulating KLF4, should be situated downstream of BMP4 pathways (Yan et al., 2016).

## BAs promote IM *via* inactivation of notch signaling pathway

A fundamental molecular signaling system that regulates cell destiny, including differentiation, proliferation, and death, is the Notch signaling pathway. Increased Notch signaling is crucial for the development of BE and has been linked to proper squamous cell differentiation and esophageal epithelial homeostasis (Kong et al., 2012). Morrow found that BAs-induced the inhibition of Notch signaling pathway, which further upregulates the expression of CDX2 and Hath1, involves in goblet cell transformation in BE (Morrow et al., 2009). In the intestine, when Notch signaling is blocked, excessive numbers of proliferative epithelial cells instantaneously are converted into goblet cells. The two opposing transcriptional factors hairy/enhancer of split 1(Hes1, also known as HRY) and atonal homolog 1 (ATOH1, also called HATH1 or Math1) are variably regulated by notch signaling, which in turn affects the direction in which intestinal progenitors differentiate (Kazanjian et al., 2010). In the intestinal epithelium of Hes1^−/−^ mice, Jensen *et al.* found that the Notch signaling pathway was lost, the number of goblet cells and endocrine cells increased and absorptive epithelial cells decreased (Jensen et al., 2000).

ATOH1 is a negative regulator in Notch signaling, in the intestine, ATOH1 is necessary for the development of the three secretory cell lineages, enteroendocrine, Paneth, and goblet cells (Yang et al., 2001). ATOH1 has been found expressed in BE and regulates the expression of the CDX2 and MUC2 (Mendelson et al., 2011). ATOH1 is a known transcriptional target of CDX2, and CDX2 activity increases the expression of the ATOH1 gene (Kong et al., 2012). In esophageal epithelial cells, Tamagawa *et al.* discovered that stimulation with BAs enhanced the expression of CDX2 and inhibited Notch signaling, enhancing ATOH1 and MUC2 expression through Hes1 repression (Tamagawa et al., 2012). Notably, Tamagawa *et al.* discovered ATOH1 to be a weaker inducer of MUC2 than CDX2, indicating that ATOH1 indirectly controls the production of MUC2 by regulating CDX2 expression (Tamagawa et al., 2012).

Delta-like 1 (Dll1), as a canonical Notch ligand, is known to play an important role in the development of IM in the small and large intestines (Tamagawa et al., 2016). Tamagawa *et al.* found that BAs enhanced ATOH1 and CDX2 expression in concentration- and time-dependent ways, while decreasing Hes1 expression in the same way. There was no difference in the levels of Notch1 mRNA expression (Tamagawa et al., 2012). Therefore, it is suggested that Dll1 does not act as a Notch agonist ligand in the canonical pathway but has a role in facilitating goblet cell metaplasia (Tamagawa et al., 2016). A study also demonstrated that BAs-induced Dll1 in BE is a CDX2-dependent process. The researchers proposed that the mRNA level of Dll1 decreased significantly after CDX2 knockdown (Tamagawa et al., 2016). In summary, Dll1 is regulated by CDX2 and Hes1 expression.

## BAs promote IM *via* EGFR signaling pathway

Dilated intercellular gaps, ongoing inflammation, and epithelial erosion are symptoms of the mucosal damage and inflammatory injuries caused by BAs in the esophagus of GERD patients. It is well known that injury to the gastrointestinal epithelium causes the epidermal growth factor receptor (EGFR) signaling to be activated as a healing mechanism. Ghatak *et al.* discovered in the 3-D culture of EPC1 primary esophageal cell line that pulse treatment BAs at pH 5 could activate the EGFR signal and the downstream effector stem ERK1/2 and Akt (Ghatak et al., 2013). Avissar *et al.* found that exposure of SEG-1 (human esophageal adenocarcinoma cells) to DCA results in activation of the EGFR and CDX2 (Avissar et al., 2009). Additionally, it has been noted that BAs binding to the gastric cancer cell line’s MBAR/TGR5 cell surface receptors promotes EGFR signaling by inducing the release of EGF ligands through the proteolytic cleavage of the pro-ligand by metalloproteinases (MP) (Yasuda et al., 2007). Matrix metalloproteinases (MMP) can be activated by BAs, particularly acidic bile. One or more dormant membrane EGFR ligands may be activated by MMP, and once they bind to EGFR, numerous tyrosine phosphorylations take place (Avissar et al., 2009). Another explanation is that other receptors may be affected by the action of BAs or acidic bile, which may activate cytoplasmic kinases and phosphorylate individual EGFR tyrosine, which has been demonstrated to function in intestinal cells (Jean-Louis et al., 2006). Alternately, EGFR activation might occur as a result of BAs producing reactive oxygen species (Casalino-Matsuda et al., 2006). In conclusion, cell signaling through the EGFR that is increased by BAs may contribute to the molecular pathogenesis of BE (Avissar et al., 2009).

## BAs promote IM *via* FXR signaling pathway

The nuclear hormone receptor superfamily’s Farnesoid X receptor (FXR), is known to play a role in the metabolism of BA, glucose, and fat. The homeostasis of BAs, including bile acid production, transport, and intestinal reabsorption, is predominantly regulated by FXR. The human gut has a high level of FXR expression, while healthy human stomach tissue has a very low level of FXR detection. FXR has a high affinity for physiological BAs. FXR has a high affinity for physiological BAs. Compared with conjugated BAs, unconjugated BAs have a stronger ability to induce FXR activation, and the strongest activating ligand for FXR is CDCA, followed by DCA, LCA, CA (Jia et al., 2018).

In healthy rat stomach epithelial cells, Xu *et al.* discovered that CDCA increases CDX2 and MUC2’s expression by activating FXR (Xu et al., 2010). In the past, it was discovered that BAs increased the expression of the FXR-target gene SHP in rat and human gastric epithelial cell lines (Park et al., 2008a; Xu et al., 2010). The nuclear receptor downstream of FXR, which plays a key role in the self-regulation of BAs metabolism, contains a particular member known as SHP. SHP-knockdown reversed the effects of CDCA on the increased protein production of CDX2 *via* the FXR pathway, as demonstrated by Zhou et al. CDCA triggered the expression of FXR, which elevated the expression of SHP at the transcriptional level (Zhou et al., 2018). According to the ChIP assay’s findings, CDCA boosted FXR’s DNA-binding activity on the SHP promoter, hence enhancing SHP expression. The chemical mechanisms by which SHP increases CDX2 expression have not yet been fully understood. As a co-regulator for target gene expression, prior research has shown that SHP interacts with NF-B in a functional way (Park et al., 2008b). A study also showed that BAs can encourage IM by increasing the expression of CDX2 and MUC2 *via* the FXR/NF-κB signaling pathway (Yu et al., 2019). FXR/SHP/NF-κB pathways may enhance BA-induced CDX2 expression based on these research results. However, the function of FXR in the digestive tract is still debatable. Inflammation-mediated damage to the gastric mucosa has been shown to be prevented by proper activation of the FXR pathway (Lian et al., 2011). A further investigation of BAs-induced FXR’s role in GIM formation is needed.

## BAs promote IM *via* microRNA

Inhibiting the expression of target genes and altering the biological activity of recipient cells, microRNAs (miRNAs) bind to the 3′-untranslated regions (3′-UTRs) of target messenger RNAs(mRNAs) to either cause their degradation or prevent their translation. They are a significant class of short non-coding regulatory RNAs (Bartel, 2009; Lujambio and Lowe, 2012). Recently, increasing evidence have identified that the dysregulated miRNA plays key roles in GIM (Sousa et al., 2016). Dysregulation of miRNAs associated with GIM found in the context of Hedgehog, NF-κB, and Wnt, which are thought to contribute to the onset and progression of GIM.

Both GC and IM patients have overexpressed miR-17–92 members (Mihailovich et al., 2015). It has been demonstrated by Li *et al.* that miR-92a-1-5p, a member of miR-17–92 cluster, was the most upregulated miRNA that induced intestine-like phenotype in gastric cells in response to BAs. It is interesting to note that the FXR antagonist glucose solution (GS) prevented the upregulation of miR-92a-1-5p in gastric cells, showing that the bile acids receptor FXR was responsible. Although c-myc, which encourages miR-17–92 transcription, was discovered to be enhanced in an FXR-independent manner by BAs feeding, BAs also control the miR-17–92 cluster through this mechanism. In addition, CDX2 expression is regulated by miR-92a-1-5p *via* the FOXD1/NF-κB pathway (Li et al., 2019).

The miR-1 level was also down in a BA-induced GIM cell model, according to a previous study (Li et al., 2019). Database analyses revealed that HDAC6 and HNF4α had the same putative miR-1 3′-UTR binding sites. Wang *et al.* revealed that by decreasing the amount of miR-1, BA induced an increase in HDAC6 and HNF4α and that the two proteins then promoted one another to establish a positive loop that eventually caused GIM (Wang et al., 2021). Both HDAC6 and HNF4α were necessary for the stomach cells to secrete mucin. A potential treatment for GIM in patients with bile reflux is the inhibition of the HDAC6/HNF4α loop and restoration of miR-1.

Intercellular communication may be a new way for BAs to promote IM (Xu et al., 2020). Exosomes play a pivotal role in intercellular communication between macrophages and gastric epithelial cells. In recipient cells, exosomal miRNAs suppress target genes through posttranslational mechanisms (Xu et al., 2020). According to a previous study, exosomes from DCA-activated macrophages carry high levels of hsa-miR-30a-5p into GES-1 cells, suppress gastric epithelial proliferation and promoted IM by targeting FOXD114 (Xu et al., 2020). Future research can start with cell-cell communication and exosomes as a therapeutic factor for IM in a BAs- related chronic inflammation microenvironment.

## Microbial communities associated with the occurrence of IM

Intestinal metaplasia occurs as a result of host-microbial interactions, and *H. pylori* infection remains a major risk factor (Zhang et al., 2005). Studies have shown that the rich intestinal microbiome is involved in the occurrence and development of intestinal metaplasia. DCA, as a representative secondary BA in the stomach, mediates IM at least at the levels of BA metabolism and microbiota. Gemobacter and *Lactobacillus* were found to be DCA-induced IM related genera. Several studies have shown that *lactobacillus* is widely colonized in human gastric mucosa (Hakalehto et al., 2011; Delgado et al., 2013). It metabolizes lactose into lactic acid, which acidifies the mucous layer of the stomach and then inhibits the secretion of gastrin and gastric acid by the G cells of the antrum (Myllyluoma et al., 2007; Jin et al., 2022). Therefore, a higher relative abundance of *Lactobacillus* in the gastric microbiome may accelerate gastric mucosal atrophy, IM, and tumorigenesis (Jin et al., 2022). In addition, a high abundance of Thermus and Anoxybacillus was also detected in gastric juices of IM patients (Liu et al., 2022). Under normal conditions, there is stable state between the microbe and the host to maintain health (Comito et al., 2014). Therefore, regulation of intestinal microecological components in IM patients by oral probiotics may be the direction of IM treatment.

## Conclusion and future perspectives

**FIGURE 1:**
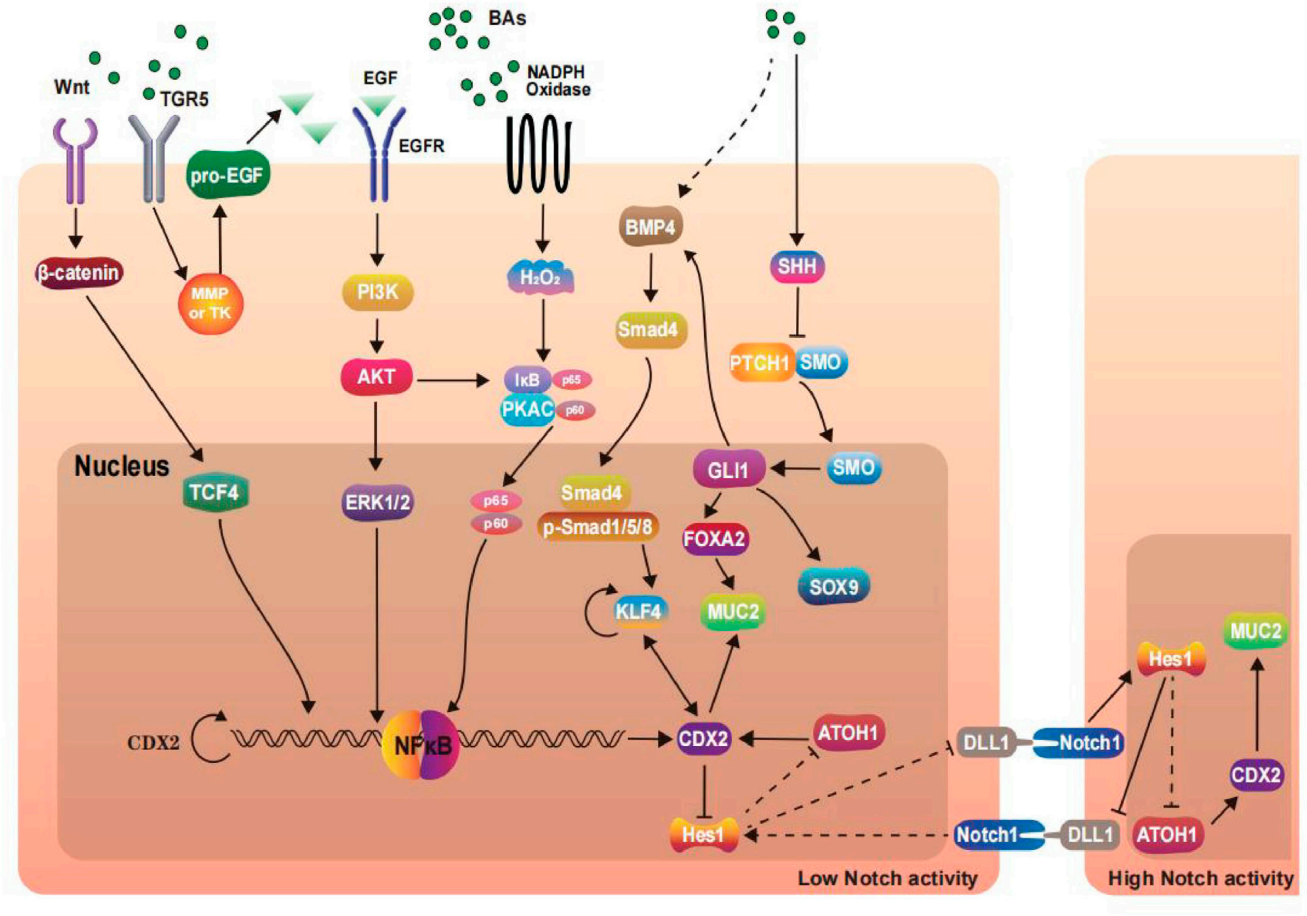
Signaling pathways and transcription factors in the development of Barrett’s esophagus.

**FIGURE 2:**
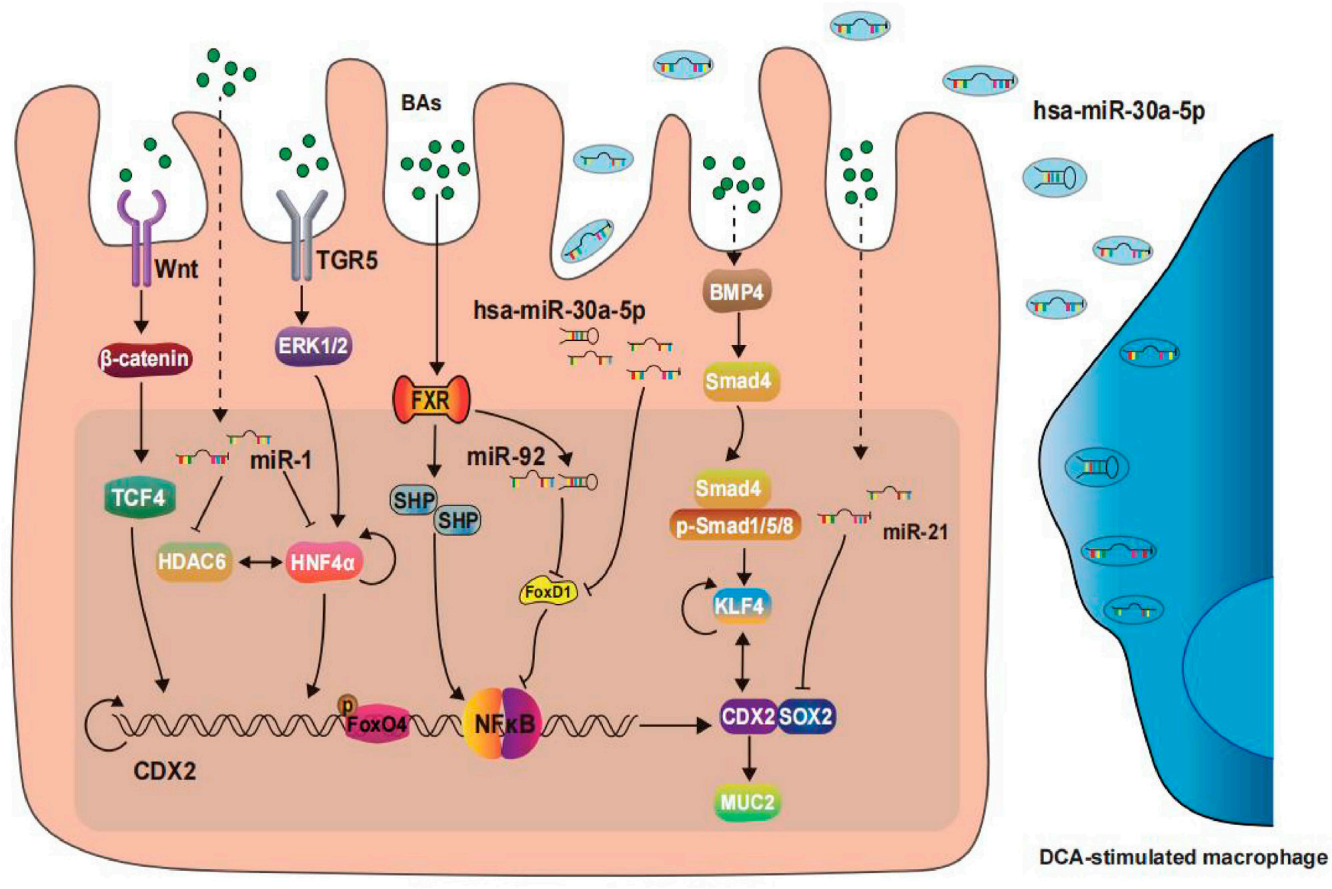
Signaling pathways and transcription factors in the development of gastric intestinal metaplasia.

The signaling pathways discussed above are depicted in Figure 1, with Figure 1 showing the BAs-induced BE pathways and Figure 2 showing the BAs-induced GIM pathways. BAs regulate the expression of CDX2 through various signal pathways, thus inducing the occurrence and development of BE and GIM. Among them, a variety of signals can trigger NF-κB activity and finally activate IκB-NF-κB-PKAc complex to regulate the transcription of CDX2. In addition, the combination of BAs and FXR induces the upregulation of miR-92a-1-5p in gastric epithelium. MiR-92a-1-5p regulates CDX2 expression through the FOXD1/NF-κB pathway, which promotes GIM progression. It is worth noting that recent studies have suggested that FOXP3, as a key transcription factor, downregulates the level of CDX2 and interacts with HDAC6 and HNF4α form HDAC6/FOXP3/HNF4α loop which participates in the development of GIM. The driver factors related with IM pathogenesis have not been completely confirmed. There is no doubt that considerable progress has been made in elucidating the pathways involved in BAs-mediated IM pathogenesis. These pathways and TFs can be modulated to provide new therapeutic opportunities. In summary, future research in BAs-induced IM should continue to concentrate on carrying out new genomic analyses, establishing more model systems and applying this knowledge to prevention and therapy.
